# The effect of GP-2250 on cultured virus-negative Merkel cell carcinoma cells: preliminary results

**DOI:** 10.1007/s00432-023-04960-3

**Published:** 2023-06-14

**Authors:** Thilo Gambichler, Britta Majchrzak-Stiller, Ilka Peters, Jürgen C. Becker, Johanna Strotmann, Nessr Abu Rached, Thomas Müller, Waldemar Uhl, Marie Buchholz, Chris Braumann

**Affiliations:** 1 Skin Cancer Center Ruhr-University, Department of Dermatology, Venereology and Allergology, Ruhr-University Bochum, Bochum, Germany; 2 Department of General and Visceral Surgery, Division of Molecular and Clinical Research, St. Josef-Hospital, Ruhr-University Bochum, 44791 Bochum, Germany; 3 Translational Skin Cancer Research, German Cancer Consortium (DKTK) Partner Site Essen/Düsseldorf, Department of Dermatology, University Duisburg-Essen, Essen, Germany; 4 Deutsches Krebsforschungszentrum (DKFZ), Heidelberg, Germany; 5 Geistlich Pharma AG, 6110 Wolhusen, Switzerland; 6 Department of General, Visceral and Vascular Surgery, Evangelische Kliniken Gelsenkirchen, Akademisches Lehrkrankenhaus der Universität Duisburg-Essen, 45878 Gelsenkirchen, Germany

**Keywords:** Merkel cell carcinoma, Merkel cell polyomavirus, Neuroendocrine tumor, Skin cancer

## Abstract

**Background:**

Even in the novel immunotherapy era, Merkel cell carcinoma (MCC) remains challenging in its treatment. Apart from Merkel cell polyomavirus (MCPyV) associated MCC, this cancer is linked in about 20% of cases to ultraviolet-induced mutational burden frequently causing aberrations in Notch and PI3K/AKT/mTOR signalling pathways. The recently developed agent GP-2250 is capable to inhibit growth of cells of different cancers, including pancreatic neuroendocrine tumors. The objective of the present study was to investigate the effects of GP-2250 on MCPyV-negative MCC cells.

**Methods:**

Methods We employed three cell lines (MCC13, MCC14.2, MCC26) which were exposed to different GP-2250doses. GP-2250’s effects on cell viability, proliferation, and migration were evaluated by means of MTT, BrdU, and scratch assays, respectively. Flow cytometry was performed for the evaluation of apoptosis and necrosis. Western blotting was implemented for the determination of AKT, mTOR, STAT3, and Notch1 protein expression.

**Results:**

Cell viability, proliferation, and migration decreased with increasing GP-2250 doses. Flow cytometry revealed a dose response to GP-2250 in all three MCC cell lines. While the viable fraction decreased, the share of necrotic and in a smaller amount the apoptotic cells increased. Regarding Notch1, AKT, mTOR, and STAT3 expression a comparatively time- and dose-dependent decrease of protein expression in the MCC13 and MCC26 cell lines was observed. By contrast, Notch1, AKT, mTOR, and STAT3 expression in MCC14.2 was scarcely altered or even increased by the three dosages of GP-2250 applied.

**Conclusions:**

The present study indicates GP-2250 having anti-neoplastic effects in MCPyV-negative tumor cells in regard to viability, proliferation, and migration. Moreover, the substance is capable of downregulating protein expression of aberrant tumorigenic pathways in MCPyV-negative MCC cells.

## Introduction

Merkel cell carcinoma (MCC) represents a rare skin cancer with epithelial as well as neuroendocrine differentiation. In approximately 80% of cases, MCPyV is clonally integrated into the tumor whereas in the remaining 20% the tumors are associated with high rates of ultraviolet (UV)-induced mutations (Becker et al. 2017a, 2022; Sihto et al. 2009; Schrama et al. 2012). It has previously been reported that MCC shows aberrations in the Notch as well as the PI3K/AKT/mTOR signalling pathways (Dobson et al. 2020; Temblador et al. 2022; Stachyra et al. 2021; Wu et al. 2021; Iwasaki et al. 2015; Lin et al. 2014). However, the exact molecular pathogenesis of this cancer and the role of MCPyV remains uncertain. MCC is a highly aggressive cancer as indicated by early relapses and very poor survival rates. Even though the treatment of advanced MCC patients has been revolutionized by the use of immune checkpoint inhibitors, there are still many patients who do not benefit from the novel immunotherapeutic approaches (Becker et al. 2017a, 2022).

Chemically, GP-2250 represents an oxathiazinane (tetrahydro-1,4,5-oxathiazine-4,4-dioxide). Buchholz et al. (Buchholz et al. 2017) published the first paper showing that GP-2250 has anti-neoplastic effects. GP-2250 represents a metabolic glyceraldehyde 3-phosphate dehydrogenase (GAPDH) inhibitor that selectively results in oxidative stress, mitochondrial dysfunction, and programmed cell death in cancer cells. In vitro as well as in vivio, GP-2250 dowregulated viability of pancreatic carcinoma cells which was associated by the induction of apoptosis and necrosis. In nude mice, GP-2250 was safe, resulting in acute (2000 mg/kg BW) or chronic (1000 mg/kg BW) toxicity only at extremely high concentrations (Buchholz et al. 2017). The same research group published further data on anti-neoplastic effects of GP-2250 on different malignancies (Braumann et al. 2020; Baron et al. 2022; Buchholz et al. 2022). In a clinical phase I/II trial on patients with advanced pancreatic carcinoma, GP-2250 is currently investigated in combination with gemcitabine (clinicaltrials.gov: NCT03854110) (Geistlich Pharma 2020). To date, the effects of GP-2250 have been studied in malignant skin cancers such as MCC and cutaneous squamous cell carcinoma (Majchrzak-Stiller et al. 2023; Barras et al. 2023). We aimed to study for the first time more in-depth the anti-neoplastic effects of GP-2250 in virus-negative MCC cell lines also addressing aberrant signalling pathways.

## Materials and methods

### Cell cultures

We studied three human MCC cell lines which were negative for MCPyV [MCC13 (CBA-1338), MCC14.2 (CBA-1340), MCC26 (CBA1341)]. On the basis of a material transfer agreement, the cell lines were provided to J.C.B. by J.H. Leonard (Queensland Radium Institute Laboratory, Queensland Institute of Medical Research, Brisbane, Australia). All cell lines have been maintained in RPMI-1640 supplemented with 10% Fetal Bovine Serum (FBS), penicillin/ streptomycin each 100 U/ml and 2 mM L-Glutamine. The culture media for MCC 14.2 and MCC 26 was additionally supplemented with 25 mM HEPES (PAN Biotech GmbH, Aidenbach, Germany). The cells were maintained as monolayer at 37 °C with 5% CO_2_ in humidified atmosphere to 60–80% confluency.

### Reagents

We used powdered GP-2250, which was provided by Geistlich Pharma AG (Wolhusen, Switzerland). The substance, dissolved in double-distilled water, was freshly prepared every other week and stored at room temperature by. It was set to a physiological pH, sterile filtered, and stored protected from light.

### MTT

The MTT assay was carried out on all cell lines in order to assess colometrically the anti-neoplastic effects of GP-2250. The MCC cells were individually seeded to obtain a sub-confluent monolayer in a 96-well plate format and were incubated for 24 h prior treatment. To examine its dose–response relationship, the MCC cells were exposed to concentrations ranging from 50 to 4500 µmol/l which depended on the cell line and ddH2O as control for 24 h. Following stated exposition times, 10 µl MTT (3-(4,5-Dimethylthiazol-2-yl)-2,5-diphenyltetra-zoliumbromid) reagent (5 mg/ml) was added and incubated for 2 h before violet Formazan crystals were dissolved in 100 µl DMSO (Dimethyl sulfoxide). The cell viability was determined by means of a microplate absorbance reader (ASYS, UVM340, Anthos Mikrosysteme GmbH, Germany) by determining the optical density at 560 nm (reference wavelength 720 nm). Using three independent experiments with consecutive passages, the MTT assay was performed with eight replicates.

### BrdU proliferation assay

The MCC cells were individually seeded to obtain a sub-confluent monolayer in a 96-well plate format and were incubated for 24 h prior treatment. To determine the dose–response of GP-2250 with respect to the anti-proliferative activity of the substance, the cells were exposed to concentrations of GP-2250 ranging from 50 to 3500 µmol/l. The exposure depended on the MCC cell line and ddH2O as control for 6 h and submitted to BrdU proliferation assay (5-bromo-2-deoxyuridine)-ELISA (Roche Applied Science, Mannheim, Germany) according to the manufacturer’s instructions. The 6-h incubation time was appropriate for the BrdU assay as indicated by our previous investigations. The amount of synthesized DNA was measured by means of a microplate absorbance reader (ASYS, UVM340, Anthos Mikrosysteme GmbH, Germany) at 450 nm with a reference wavelength of 550 nm*.* Using three independent experiments with consecutive passages*,* BrdU assay was performed with eight replicates.

### Scratch assay

To form a confluent monolayer, all three MCC cell lines were plated into 60 mm dishes and incubated for 24 h. The needed number of cells for a confluent monolayer was dependent on the MCC cell type. All cell lines were exposed to GP-2250 concentrations ranging from 250 up to 4000 µmol/l. After introducing a scratch in the cell monolayer, mimicking a wound, the area was examined by phase-contrast-microscopy. Images of the scratch assay were captured at start of the exposures and at intervals (0, 6, 12, and 24 h) during the migration of cells closing the scratch, to semi-quantify the migration rates.

### Flow cytometry analysis

Again, the MCC cells were individually seeded in order to obtain a sub-confluent monolayer in a 6-well plate format and were incubated for 24 h. Different individual concentrations of GP-2250 (from 100 up to 4000 μmol/l) and ddH2O (control) were utilized for 24 h before the cells were assessed by flow cytometry analysis. The latter was carried out in 4 to 6 independent measurements including 2 to 4 consecutive passages. The MCC sells were resuspended in a 200 μl binding buffer (Bender MedSystems, Vienna, Austria). Thereafter, the cell suspension was treated with 5 to 10 μl Annexin V-FITC (BD Biosciences, Heidelberg, Germany) over 15 min at room temperature in the dark. Then, 10 μl of propidium iodide (PI) (Bender MedSystems, Vienna, Austria) were added. Subsequently, the cells were assessed by flow cytometry (FACS Celesta BD Biosciences, Heidelberg, Germany) for Annexin V-FITC and PI binding. The dot plots and histograms were evaluated by means of the CellQuest Pro software (BD Biosciences, Heidelberg, Germany).

### Western blot analysis

Protein isolation was performed by RIPA (Radio immunoprecipitation assay) lysis (Abcam, Cambridge, UK). Subsequently a BCA (Bicinchoninic acid) assay (Thermo scientific, IL, USA) was used for protein quantification. After loading equal amounts of protein per lane (30 μg protein), 7 to 20% Protean-TGX (Tris–Glycine eXtended) gels (BIO RAD, Hercules, California, USA), were electrophoresed at 250 V for 30 to 45 min and transferred on to a TransBlot Turbo PVDF (Polyvinylidene fluoride) membrane (BIO RAD, Hercules, California, USA) using a TransBlot Turbo system (BIO RAD, Hercules, California, USA). In line with the manufacturer’s antibody specification protocol, the membranes were blocked in EveryBlock Blocking buffer (BIO RAD, Hercules, California, USA) over 5 min and incubated overnight at 4 °C with primary antibody (mTOR Rabbit Ab #2972, AKT Rabbit Ab #9272, beta-Actin Rabbit mAB #8457, HSP 90 Rabbit AB #4874, Notch1 Rabbit mAB #3608, STAT3 Rabbit mAB #12,640) at 1:1000 dilution. Thereafter, the membranes were washed using PBST (phosphate buffered saline + tween 0.025%) and incubated with an Anti-rabbit IgG HRP-linked AB 7074; (1:2000 CST, Denver, Massachusetts, USA). Band detection was carried out using ChemiDoc MP imaging system (BIO RAD, Hercules, California, USA). Comparative quantification of Western blot results was performed using the BIO RAD Image lab software, Version 6.1, (BIO RAD, Hercules, California, USA).

### Statistics

The data of the MTT assay (%, viable cells), BrDU assay (%, proliferating cells), scratch assay (%, wound area), and flow cytometry analysis (%, apoptotic, necrotic, and vital cells) are given in mean ± SD. We used ANOVA including the Tukey’s post-hoc test for normally distributed data. Where appropriate, the *t* test was employed for pairwise comparisons. *p* values ≤ 0.05 were considered statistically significant and are shown in the figures as follows: ***p ≤ 0.001, **p ≤ 0.01, *p ≤ 0.05. Statistics were performed by means of Graph Pad Prism 9.1.0 (Graph Pad Software, San Diego, USA).

## Results

**Fig. 1:**
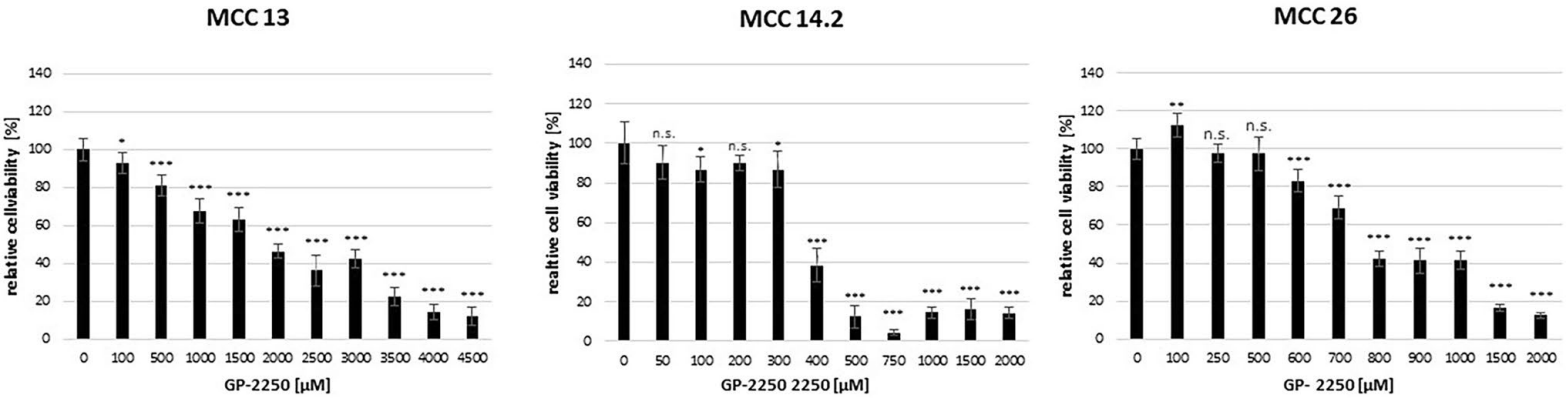
Demonstrating that all MCC cell lines showed a good dose response to GP-2250 during MTT viability assay to different extends. MCC 13, MCC 14.2, MCC26, cells were incubated with individual concentrations of GP-2250 and ddH2O for 24 h and submitted to a MTT-assay. Values are means ± SEM of 8 replicates of three independent experiments with consecutive passages. Asterisk symbols indicate differences between control, which was adjusted to 100% and 2250 treatment. ***p ≤ 0.001, **p ≤ 0.01, *p ≤ 0.05, n.s. *p* > 0.05 (one-way ANOVA followed by Tukey’s post-hoc test)

Figure 1 illustrates a cell line dependent GP-2250 dose response in all three MCC lines during MTT viability assay, demonstrating its cytotoxic effects on MCC. MCC 13 exhibited ad classic dose response with a relative high tolerability towards the substance (EC 50 approximately between 2000 and 3000 µM GP-2250), whereas MCC14.2 showed a very sharp viability profile between 300 µM and 400 µM resulting in a drop of viability from 86.6 ± 9.4% to 38.5 ± 8.8%. By contrast, MCC 26 displayed a classic dose response as well, with an effective dosage ranging from 600 µM GP-2250 up to a maximum of 2000 µM.

**Fig. 2:**
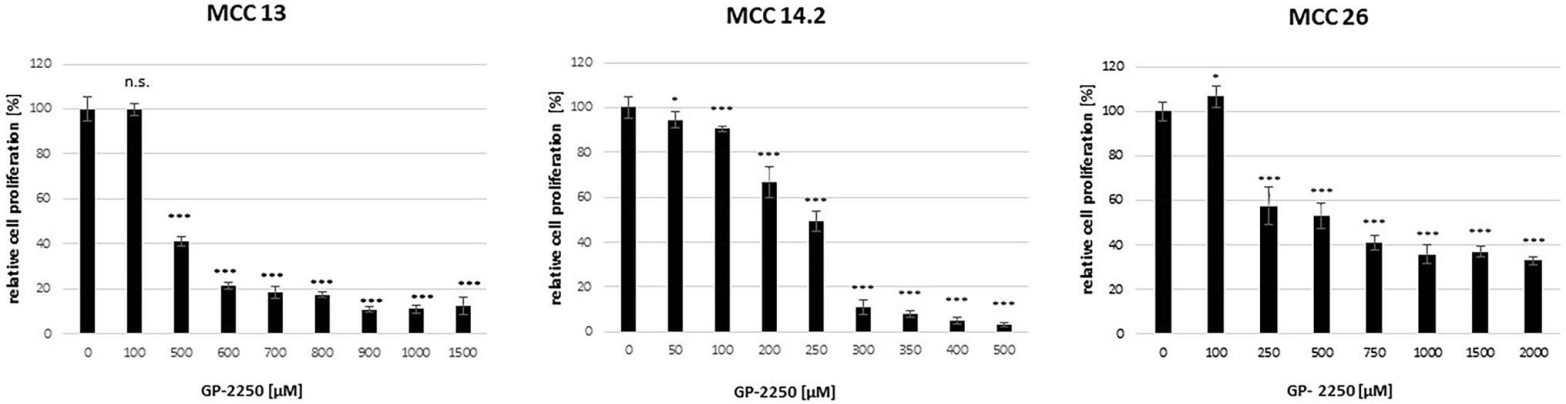
Showing that all three MCC cell lines showed a dose response to GP-2250 during BrDU proliferation assay to different extends. MCC 13, MCC 14.2, MCC26, cells were incubated with individual concentrations of GP-2250 and ddH2O for 24 h and submitted to a BrDU-assay. Values are means ± SEM of 8 replicates of three independent experiments with consecutive passages. Asterisk symbols indicate differences between control, which was adjusted to 100% and 2250 treatment. ***p ≤ 0.001, **p ≤ 0.01, *p ≤ 0.05, n.s. *p* > 0.05 (one-way ANOVA followed by Tukey’s post-hoc test)

Correspondingly, all three MCC cells demonstrated a dose response to GP-2250 during BrDU to different range (Fig. 2), thus demonstrating the anti-proliferative effects of GP-2250 on MCC cells. In contrast to the data obtained from the MTT assay, MCC 13 exhibited an effective inhibition of cell proliferation to comparative low dosages starting from 500 µM GP-2250 on. (Fig. 2). Again, MCC14.2 showed a sharp profile of proliferation inhibition between 250 µM and 300 µM resulting in a drop of proliferation from 49.3% ± 4.3% to 10.9% ± 3.1%. MCC26 presented a classic dose response including a comparable high residual proliferation rate at higher concentrations with a rate of 32.9% ± 1.9% under the influence of 2000 µM GP-2250.

**Fig. 3:**
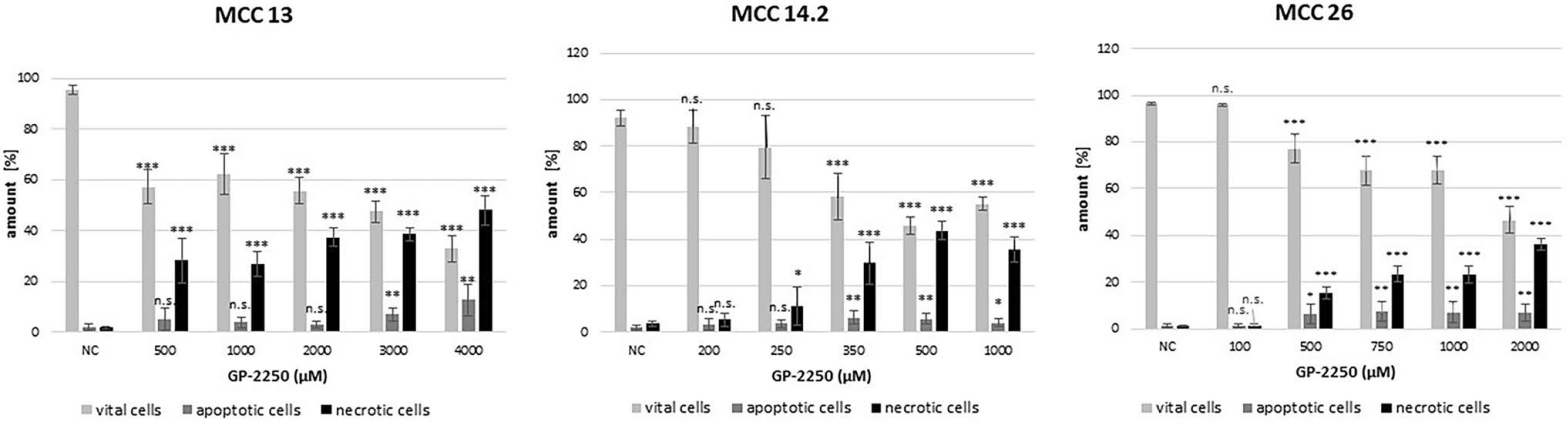
Flow cytometry for evaluation of apoptosis and necrosis revealed that all MCC cell lines showed a dose response to GP-2250, which was comparable to the dosages used for the MTT assay (Fig. 1). MCC 13, MCC 14.2, MCC26, cells were incubated with individual concentrations of GP-2250 and ddH2O for 24 h. The percentages of viable, apoptotic and necrotic cells were determined by FACS-analysis with Annexin V-FITC and propidium iodide. Values are means ± SEM of 4–6 independent experiments with three consecutive passages. Asterisk symbols on columns indicate differences between control and 2250 treatment. ***p ≤ 0.001, **p ≤ 0.01, *p ≤ 0.05, n.s. *p* > 0.05 (one-way ANOVA followed by Tukey’s post-hoc test)

Concerning flow cytometric measurements, all three MCC cell lines showed a dose response to GP-2250, its range varying between cell lines (Fig. 3). Flow cytometry showed the effect of GP-2250 on apoptosis as well as necrosis. Response rates and dosages were comparable to those used for the MTT viability assay previously. Overall, in all cell lines assessed, the rate of viable cells decreased, simultaneously the necrotic and, to a smaller extent, the apoptotic fraction increased with rising concentrations of GP-2250. While MCC13 was comparable resistant, with 32.9% ± 3.2% viable cells at a concentration of 4000 µM GP-2250, MCC14.2 and MCC26 reacted more sensitively towards the treatment with GP-2250 (MCC 14.2: 55.1 ± 2.7% at 1000 µM; MCC 26: 46.6 ± 5.7% at 2000 µM).

**Fig. 4:**
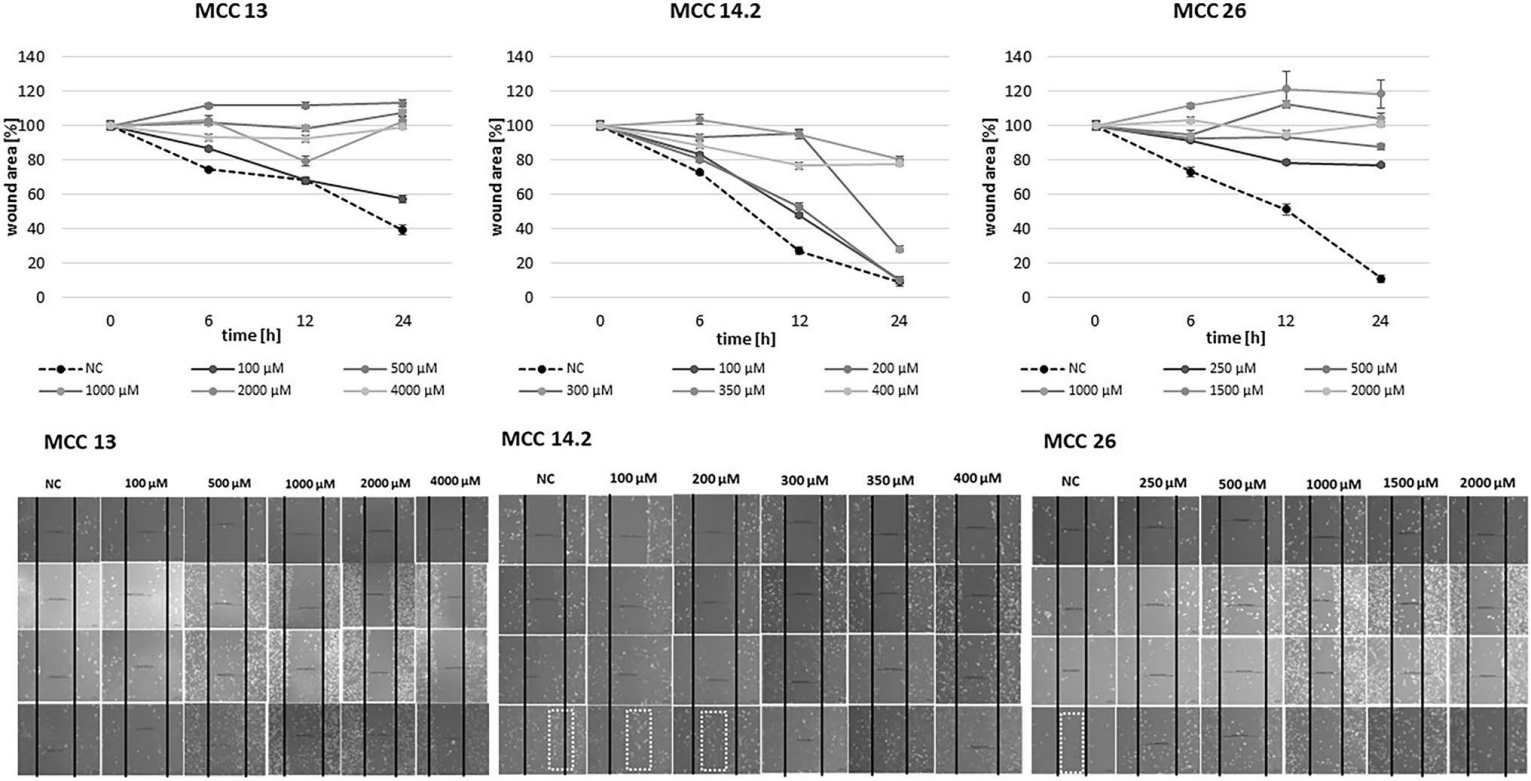
The scratch assay revealed that all MCC cell lines show a dose response to GP-2250 to different extends. A confluent monolayer of MCC 13, MCC 14.2, MCC26, cells with an introduced scratch were incubated with individual concentrations of GP-2250 and ddH2O an microscopically monitored for 24 h with measurements at 0, 6, 12 and 24 h. Values are means ± SEM of 3 independent measurements

As displayed in Fig. 4, all MCC cell lines showed a dose response to GP-2250 during scratch assay. Additionally, readings over 24 h reveal the impact of GP-2250 on the migration rate of the MCC cell lines analyzed. The results demonstrate similar effective concentrations between 350 µM and 500°µM in all cell lines (in MCC 13 and MCC 26: starting at 500 µM; in MCC 14.2 at 350 µM).

**Fig. 5:**
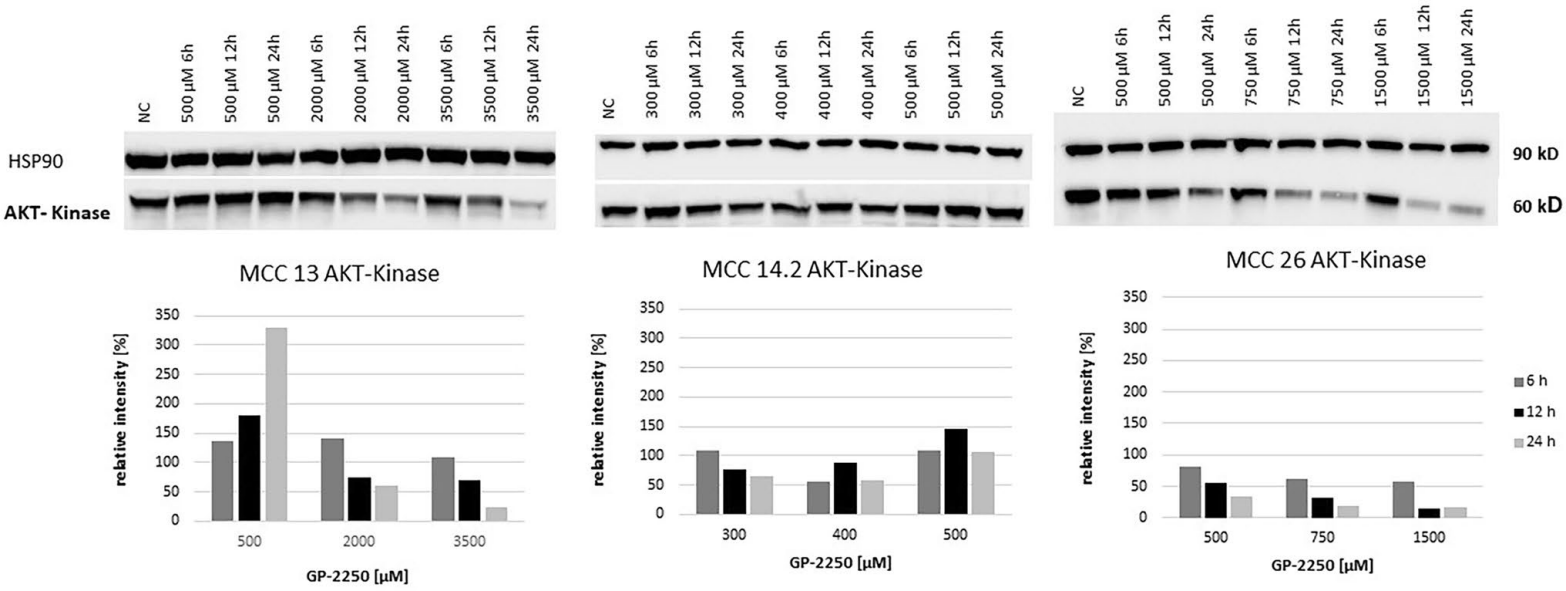
Western blot and quantitative analyisis of AKT kinase protein expression (*n* = 1). Data presented at three independent time points (6 h, 12 h, 24 h) in three Merkel carcinoma cell lines MCC13, MCC14.2, and MCC26 after treatment using three individual dosages of GP-2250. HSP-90 was used as internal control

Western blotting was performed for protein expression analysis of AKT, mTOR, STAT3, and Notch1 in all three MCC cell lines. Cells were exposed to GP-2250 applied in three individual concentrations, having proven effective in previous experiments. Regarding AKT expression, there was a moderate time- and dose-dependend decrease of protein expression in MCC13 and MCC26 (Fig. 5). AKT expression in MCC14.2 was scarcely altered by the three applied dosages of GP-2250.

**Fig. 6:**
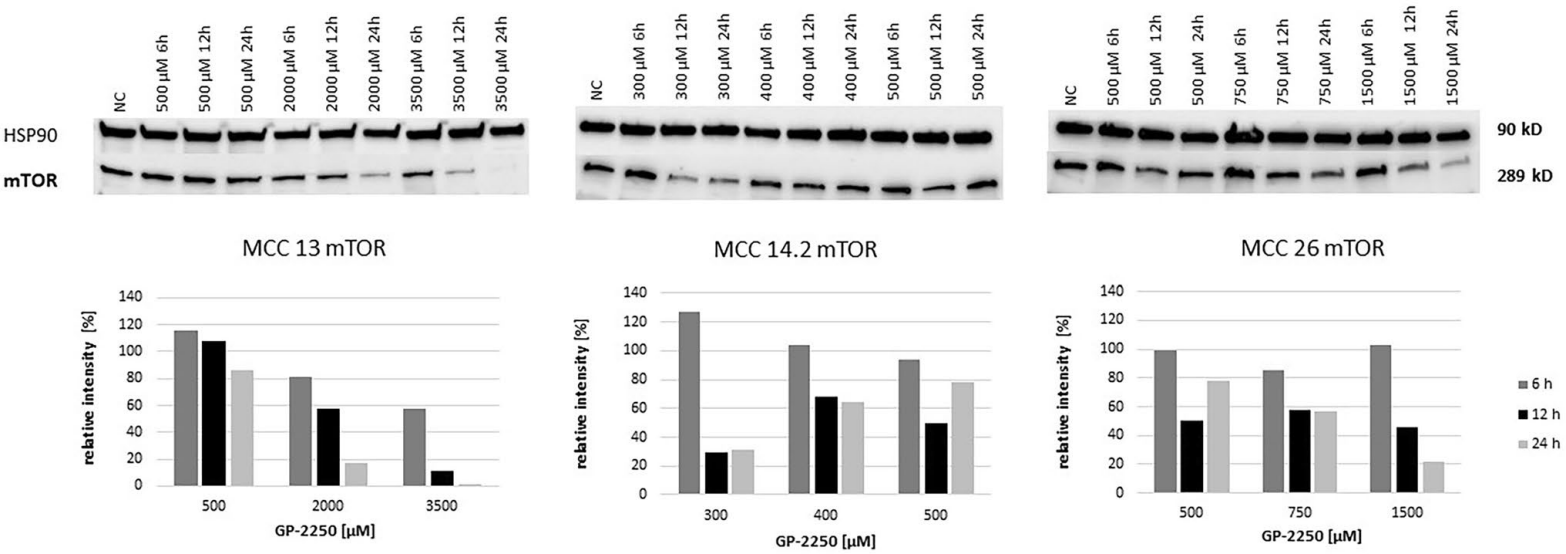
Western blot and quantitative analyisis of mTOR kinase protein expression (*n* = 1). Data presented at three independent time points (6 h, 12 h, 24 h) in three Merkel carcinoma cell lines MCC13, MCC14.2, and MCC26 after treatment using three individual dosages of GP-2250. HSP-90 was used as internal control

**Fig. 7:**
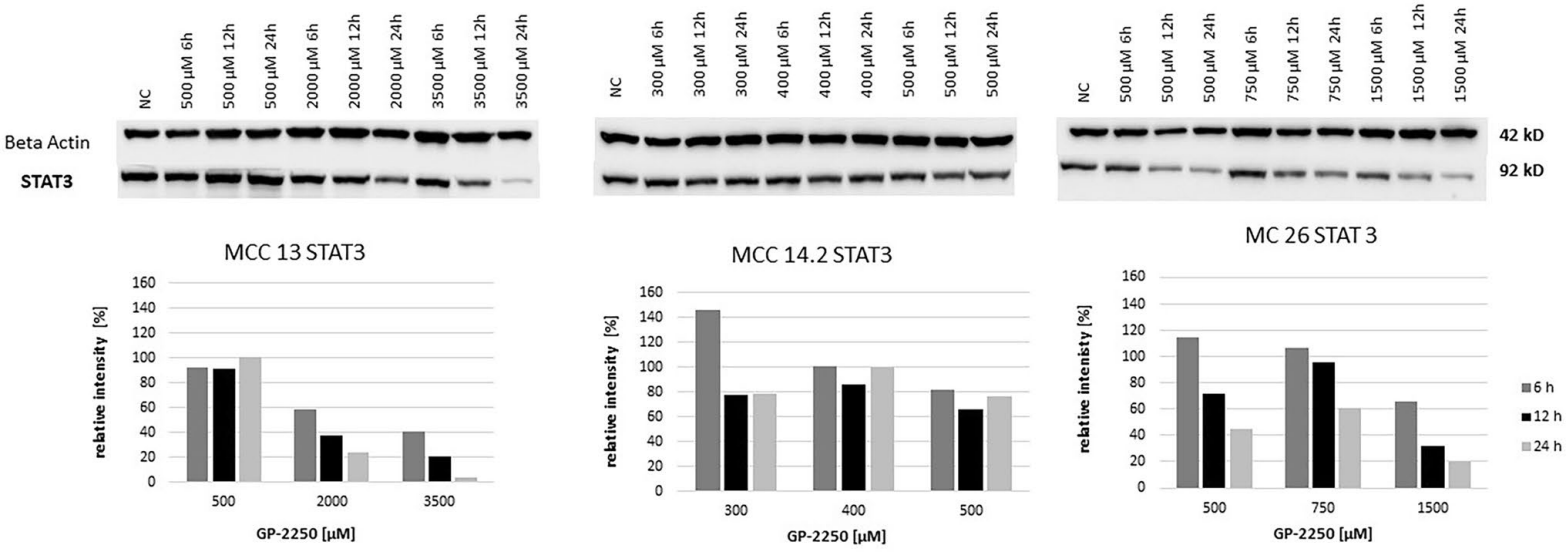
Western blot and quantitative analyisis of STAT3 kinase protein expression (*n* = 1). Data presented at three independent time points (6 h, 12 h, 24 h) in three Merkel carcinoma cell lines MCC13, MCC14.2, and MCC26 after treatment using three individual dosages of GP-2250. HSP-90 was used as internal control

As shown in Fig. 6, mTOR protein expression was substantially decreased in MCC13 and MCC26 cell lines after 24 h and the application of the highest GP-2250 doses. By contrast, higher doses of GP-2250 applied to MCC14.2 appeared to increase mTOR protein expression, in particular at 24 h. Regarding STAT3 expression, protein expression of MCC13 and MCC26 was reduced in a time- and dose-dependent manner (Fig. 7). Again, STAT3 expression in MCC14.2 was only slightly altered by the three GP-2250 dosages used.

**Fig. 8:**
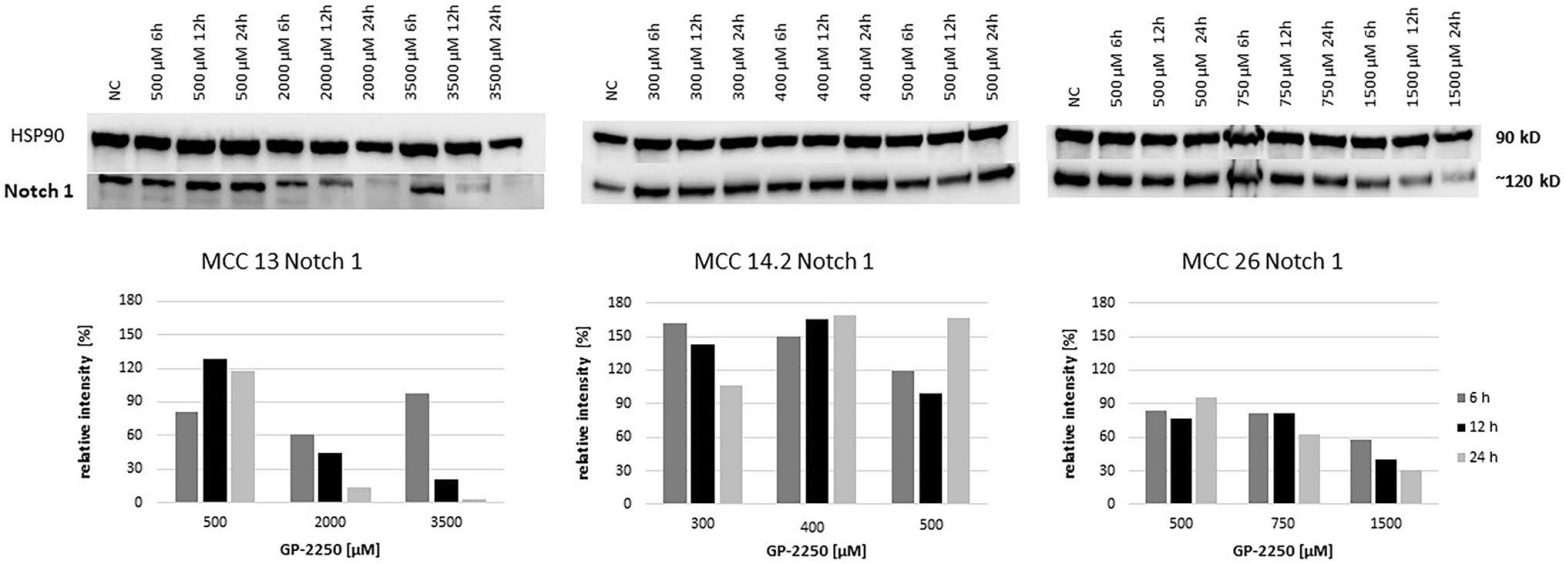
Western blot and quantitative analyisis of Notch1 kinase protein expression (*n* = 1). Data presented at three independent time points (6 h, 12 h, 24 h) in three Merkel carcinoma cell lines MCC13, MCC14.2, and MCC26 after treatment using three individual dosages of GP-2250. HSP-90 was used as internal control

Figure 8 displays the protein expression of Notch1 indicating that the expression is dimished in a rather time- and dose-dependent pattern in MCC13 and MCC26 cell lines, whereas the Notch1 expression profile in MCC14.2 was rather inconsistent in respect to time and dose effects.

## Discussion

The mutational tumor burden of MCC differs significantly between MCPyV-negative MCC and MCPyV-positive MCC. Virus-negative MCC has a very high overall mutational rate when compared to virus-positive MCC (Stachyra et al. 2021; Wong et al. 2015; Goh et al. 2016; Panelos et al. 2009). Hence, it is not surprising that the intra-tumoral T cell receptor repertoire in virus-positive MCC is clonal whereas virus-negative MCC is characterized by a diverse T cell receptor reservoir. Virus-negative MCC is characterized by a high number of UV-induced DNA mutations being 90-fold increased compared to virus-positive MCC (Stachyra et al. 2021; Wardhani et al. 2019; Brazel et al. 2023; Horny et al. 2021; Harms et al. 2015; Becker et al. 2017b). Apart from *RB1* and *TP53* mutations*,* aberrations occur also frequently in *Notch* genes as well as in the PI3K/AKT/mTOR signaling pathway which we have addressed in the present study (Dobson et al. 2020; Temblador et al. 2022; Stachyra et al. 2021; Wu et al. 2021; Iwasaki et al. 2015; Lin et al. 2014). Previous findings suggest that MCC pathogenesis may be molecularly divided into virus-induced and UV-induced etiologies, while virus-negative MCC patients have a threefold lower 5-year survival rate than patients with virus-positive MCC (Becker et al. 2017b).

Notch1 signaling plays a significant role in differentiation, proliferation, cell fate, and programmed cell death. By contrast, signaling of Notch2 takes part both in cell fate determination in the embryo and regulation of the immune system. Both pathways have been shown to be affected in MCC. About ¾ of virus-negative MCC show alterations in *Notch*, thus *Notch1* mutations are observed in up to 90% of MCC cases (Goh et al. 2016; Panelos J et al. 2009; Wardhani et al. 2019; Brazel et al. 2023). Again, most mutations in MCC are characterized by UV signatures (Horny et al. 2021; Harms et al. 2015). Furthermore, the PI3K/AKT/mTOR pathway is upregulated in MCC. PI3K plays a significant role in cell growth, proliferation, migration, and protein translation. Thus, the PI3K/AKT signaling is crucial for the survival of cells. Furthermore, *AKT1*gain-of-function mutants have previously been observed. There is are interactions between the AKT/mTOR signaling and several other pathways, such as the JAK‐STAT signaling pathway. Consequently, the activation of JAK2 results in activation of STAT transcription factors (Dobson et al. 2020; Temblador et al. 2022; Stachyra et al. 2021; Wu et al. 2021; Iwasaki et al. 2015, 2022; Lin et al. 2014; Harms et al. 2015; Becker et al. 2017b; Guo et al. 2013).

The exact mode of action of GP-2250 remains yet to be fully established. However, GP-2250 is capable to deplete metabolic energy through inhibition of the enzyme GAPDH which is rate limiting for aerobic glycolysis (Braumann et al. 2020). Overexpression of GAPDH has been reported in a variety of cancers and is associated with a poor survival rate. As a result, GAPDH inhibition has been identified as a promising strategy for the treatment of cancer and other conditions. Moreover, GAPDH inhibitors are capable to influence mTOR and AKT mediated responses via changes in TORC1 activity (Guo et al. 2013). Previous data of our study group, however, indicate that cell death induction through enhanced release both of ROS and mitochondrial alterations may play a major role (Buchholz et al. 2017, 2022; Braumann et al. 2020; Baron et al. 2022). In animal models, GP-2250 showed a decrease of growth of cancer cells both in xenografts of pancreatic cancer and xenograft models of pancreatic adenocarcinoma while being only infrequently associated with adverse events. In nude mice, there were no body weight alterations or organ dysfunctions at applied concentrations (Braumann et al. 2020). Furthermore, GP-2250 has also been tested combined with chemotherapeutic substances, including gemcitabine, cisplatin and mitomycin C. Several experiments in malignant peritoneal mesothelioma, pancreatic adenocarcinoma, and neuroendocrine pancreatic carcinoma suggest synergistic effects between the substance GP-2250 and gemcitabine or cisplatin and mitomycin C respectively (Braumann et al. 2020; Baron et al. 2022; Buchholz et al. 2022). In neuroendocrine carcinomas of the pancreas, the first-choice treatments are platin-based agents combined with etoposide which are characterized by poor very tolerability (Buchholz et al. 2022). Prior to the immunotherapy era, these agents were also used first-line in metastatic MCC (Iwasaki et al. 2022). In the present study, all MCC cell lines showed a cell line dependent dose response to GP-2250 during MTT, BrDU, and scratch tests. Cell viability, proliferation, and migration decreased with increasing GP-2250 doses. In this regard, the present data give support to previous studies on the use of GP-2250 in other malignancies (Buchholz et al. 2017, 2022; Braumann et al. 2020; Baron et al. 2022). Flow cytometry revealed a dose response to GP-2250 in all three MCC cell lines which was comparable to the dosages used for the MTT assay. While the viable fraction decreased, the necrotic fraction and in a smaller amount the apoptotic fraction increased. Western blotting was performed for protein expression analysis of parts of the Notch and PI3K/AKT/mTOR pathways in MCC cells investigated. The cells were exposed to GP-2250 using three concentrations. With regard to Notch1, AKT, mTOR, and STAT3 expression there was a comparatively time- and dose-dependend decrease of protein expression in the MCC13 and MCC26 cell lines. In MCC 14.2 expression of mTOR was likewise decreased. By contrast, AKT and STAT3 expression in MCC14.2 was hardly altered or even increased by the three dosages of GP-2250 applied.

Indeed, limitations of the present study include the divergent findings of western blotting between MCC14.2 and the other two cell lines. This discrepancy can only be in parts explained by differences with respect to underlying tumor characteristics. The cell line MCC14.2 originates from an iliac lymph node metastasis harboring a *TP53* mutation, whereas MCC13 originates from a cervical lymph node metastasis with *TP53* mutations and MCC26 is presumably derived from a cutaneous primary tumor harboring *HRAS* and *RB1* mutations (https://www.cellosaurus.org, access: January 22, 2023; Gravemeyer et al. 2021). We can only speculate that MCC14.2 tumor cells have other mutations, which might explain the differences in the effects observed following GP-2250 treatment. Possibly, there is a link between the underlying mutations and the mTOR, AKT, and STAT3 pathways. Nevertheless in at least two MCC cell lines, the protein expression of Notch1, AKT, mTOR, and STAT3 was downregulated by GP-2250, thus indicating that altered Notch and PI3K/AKT/mTOR pathways in virus-negative MCC can effectively be addressed by the novel substance GP-2250.

## Conclusions

Our preliminary data indicate that GP-2250 has anti-neoplastic effects in virus-negative MCC cells regarding tumor cell viability, proliferation, and migration. Moreover, GP-2250 is capable to downregulate protein expression of aberrant tumorigenic pathways in virus-negative MCC cell lines.

## Data Availability

The datasets used and analyzed during the current study are available from the corresponding author on reasonable request.
